# Supplementary material for: Nutrigenomic Effect of Saturated and Unsaturated Long Chain Fatty Acids on Lipid-Related Genes in Goat Mammary Epithelial Cells: What Is the Role of PPARγ?
Source: Vet Sci. 2019 Jun 11;6(2):54. doi: 10.3390/vetsci6020054 (PMC6632130; doi:10.3390/vetsci6020054)
Supplement: Supplementary file 1 [file vetsci-06-00054-s001.zip › vetsci-497288-SI/Table S1.docx]

**Table S1** Primer-pairs features, Table S2. Gene symbol, description, and biological process of genes investigated

| Accession# | Gene | Primers (5’-3’) | Product Size (bp) |
| --- | --- | --- | --- |
| JN236219.1 | *ACACA* | Foward: CTCCAACCTCAACCACTACGG  Reverse: GGGGAATCACAGAAGCAGCC | 171 |
| XM_004021695.1 | *ACSL1* | Foward: GTGGGCTCCTTTGAAGAACTAT  Reverse: ATAGATGCCTTTGACCTGTTCAAAT | 120 |
| JI861797.1 | *AGPAT6* | Foward: AAGCAAGTTGCCCATCCTCA  Reverse: AAACTGTGGCTCCAATTTCGA | 101 |
| XM_005675092.1 | *BDH1* | Foward: CCCGCCACGAGTCTGAGCAT  Reverse: CCCACGACTCTGCACCCCAA | 101 |
| NM_001285578.1 | *CD36* | Foward: GTACAGATGCAGCCTCATTTCC  Reverse: TAGACCTGCAAATATCAGAGGA | 81 |
| DQ380249.1 | *DGAT1* | Foward: CCACTGGGACCTGAGGTGTC  Reverse: GCATCACCACACACCAATTCA | 101 |
| NM_001009350 | *FABP3* | Foward: GATGAGACCACGGCAGATG  Reverse: GTCAACTATTTCCCGCACAAG | 120 |
| JQ031288.1 | *FABP4* | Foward: TACCTGGAAACTTGTCTCC  Reverse: CTGATTTAATGTTGACCACAT | 145 |
| XM_005699765.1 | *FADS1* | Foward: GGTGGACTTGGCCTGGATG  Reverse: TGACCATGAAGAAAAGCCCC | 101 |
| DQ915966.3 | *FASN* | Foward: GGGCTCCACCACCGTGTTCCA  Reverse: GCTCTGCTGGGCCTGCAGCTG | 226 |
| AJ431207 | *GAPDH* | Foward: GCAAGTTCCACGGCACAG  Reverse: GGTTCACGCCCATCACAA | 249 |
| XM_004020197.1 | *GPAM* | Foward: ACCAGCAGTTCATCACCTTC  Reverse: GTACACGGCAACCCTCCTCT | 170 |
| XM_004008186.1 | *INSIG1* | Foward: TAAGCCTCGAACTAAAGCCTAACT  Reverse: TTCCTATCTCACCACACTTCATCT | 101 |
| JX392387.1 | *LPIN1* | Foward: TGGCCACCAGAATAAAGCATG  Reverse: GCTGACGCTGGACAACAGG | 101 |
| JQ670882.1 | *LPL* | Foward: AGGACACTTGCCACCTCATTC  Reverse: TTGGAGTCTGGTTCCCTCTTGTA | 169 |
| XM_004022004.1 | *MID1IP1* | Foward: ATGTCGGCGTGGAGGTAGG  Reverse: CGATGTCGTTGCGGATGG | 153 |
| XM_005693601.1 | *NCOR1* | Foward: TGCAAGAAAACAAAGGGAACAA  Reverse: CCTCCGAGGGTTATTTTCTATT | 100 |
| GU332719 | *NR1H3* | Foward: CATCAACCCCATCTTCGAGTT  Reverse: CAGGGCCTCCACATATGTGT | 163 |
| XM_005694769.1 | *OXCT1* | Foward: CAATGCTAGGAGCCATGCAG  Reverse: CACTAGATCCATAGCCCCTCCC | 101 |
| HQ846826 | *PLIN2* | Foward: TACGATGATACAGATGAATCCCAC  Reverse: CAGCATTGCGAAGCACAGAGT | 203 |
| XM_004018768.1 | *PPARD* | Foward: CATGTGGCAGCCTCAACATG  Reverse: GACGGAAGAAGCCCTTGCA | 102 |
| HQ589347.1 | *PPARG* | Foward: CCTTCACCACCGTTGACTTCT  Reverse: GATACAGGCTCCACTTTGATTGC | 145 |
| XM_005709411.1 | *RPS9* | Foward: CCTCGACCAAGAGCTGAAG  Reverse: CCTCCAGACCTCACGTTTGTTC | 64 |
| XM_002691687.2 | *RXRA* | Foward: CGCTCCTCAGGCAAGCA  Reverse: TGTCAATCAGGCAGTCCTTGTT | 121 |
| XM_005696121.1 | *SCAP* | Foward: CCATGTGCACTTCAAGGAGGA  Reverse: ATGTCGATCTTGCGTGTGGAG | 108 |
| GU947654 | *SCD1* | Foward: CCATCGCCTGTGGAGTCAC  Reverse: GTCGGATAAATCTAGCGTAGCA | 257 |
| NM_001033625.2 | *SLC27A1* | Foward: GGCAAGGGCATGGATGATC  Reverse: CCCGTGGTACCTGCTGTGCAC | 96 |
| NM_001285755.1 | *SREBF1* | Foward: GTGCTGAGGGCAGAGATGGC  Reverse: ACAAAGAGAAGCGCCAAGGAGAA | 106 |
| XM_005681190.1 | *SREBF2* | Foward: GGGCAGCAGAGTTCTTTCTG  Reverse: GCTGGACCACATGGTTAAGGTC | 109 |
| XM_005700842.1 | *UXT* | Foward: TGTGGCCCTTGGATATGGTT  Reverse: GGTTGTCGCTGAGCTCTGTG | 101 |
| XM_005683765.1 | *VLDLR* | Foward: GCCCAGAACAGTGCCATATGA  Reverse: TTTTCACCATCGCACCGCC | 100 |
