# Supplementary material for: Nutrigenomic Effect of Saturated and Unsaturated Long Chain Fatty Acids on Lipid-Related Genes in Goat Mammary Epithelial Cells: What Is the Role of PPARγ?
Source: Vet Sci. 2019 Jun 11;6(2):54. doi: 10.3390/vetsci6020054 (PMC6632130; doi:10.3390/vetsci6020054)
Supplement: Supplementary file 1 [file vetsci-06-00054-s001.zip › vetsci-497288-SI/Table S2.docx]

**Table S2** Gene symbol, description, and biological process of genes investigated as reported in Entrez Gene, National Center for Biotechnology Information (NCBI).

| **Gene symbol** | **Description** | **Biological process** |
| --- | --- | --- |
| *ACACA* | Acetyl-coenzyme A carboxylase | Fatty acid biosynthesis |
| *ACSL1* | Acyl-coenzyme A synthetase long-chain family member 1 | Fatty acid metabolism |
| *AGPAT6* | 1-acylglycerol-3-phosphate O-acyltransferase 6 | Phosphatidic acid biosynthesis |
| *BDH1* | 3-hydroxybutyrate dehydrogenase 1 | Ketone body utilization |
| *CD36* | The fatty acid translocase CD36 molecule [thrombospondin receptor] | Fatty acid metabolism |
| *DGAT1* | Diacylglycerol O-acyltransferase homolog 1 | Triacylglycerol metabolism |
| *FABP3* | Fatty acid-binding protein 3 | Fatty acid binding, transport |
| *FABP4* | Fatty acid-binding protein 4 | Fatty acid binding, transport |
| *FADS1* | Fatty acid desaturase 1 | Unsaturated fatty acid synthesis |
| *FASN* | Fatty acid synthetase | Fatty acid biosynthesis |
| *GPAM* | Glycerol-3-phosphate acyltransferase, mitochondrial | Triacylglycerol biosynthesis |
| *INSIG1* | Insulin-induced gene 1 | Lipid metabolism, cell proliferation |
| *LPIN1* | Lipin 1 | Phosphatidic acid hydrolysis, transcription |
| *LPL* | Lipoprotein lipase | Triacylglycerol metabolism |
| *NR1H3* | Liver X receptor alpha | Transcription regulation |
| *MID1IP1* | Mid1 interacting protein 1 | Triacylglycerol synthesis and lipid droplet formation |
| *NOR1* | Neuron-derived orphan receptor 1 | Transcription regulation |
| *OXCT1* | 3-oxoacid coa-transferase 1 | Ketone body utilization |
| *PLIN2* | Adipose differentiation-related protein | Triacylglycerol synthesis and lipid droplet formation |
| *PPARD* | Peroxisome proliferator activated receptor delta | Transcription regulation |
| *PPARG* | Peroxisome proliferator-activated receptor gamma | Induction of adipocyte differentiation, transcription |
| *RXRA* | Retinoid x receptor alpha | Transcription regulation |
| *SCAP* | SREBF chaperone | SREBF target gene transcription activation |
| *SCD1* | Stearoyl-coenzyme A desaturase 1 | Fatty acid synthesis and desaturation |
| *SLC27A1* | Long-chain fatty acid transport protein 1 | Fatty acid transport |
| *SREBF1* | Sterol regulatory element-binding transcription factor 1 | Transcription regulation |
| *SREBF2* | Sterol regulatory element-binding transcription factor 2 | Transcription regulation |
| *VLDLR* | Very-low-density-lipoprotein receptor | Fatty acid import into cells |
