# Supplementary figures and images for: Nutrigenomic Effect of Saturated and Unsaturated Long Chain Fatty Acids on Lipid-Related Genes in Goat Mammary Epithelial Cells: What Is the Role of PPARγ?
Source: Vet Sci. 2019 Jun 11;6(2):54. doi: 10.3390/vetsci6020054 (PMC6632130; doi:10.3390/vetsci6020054)

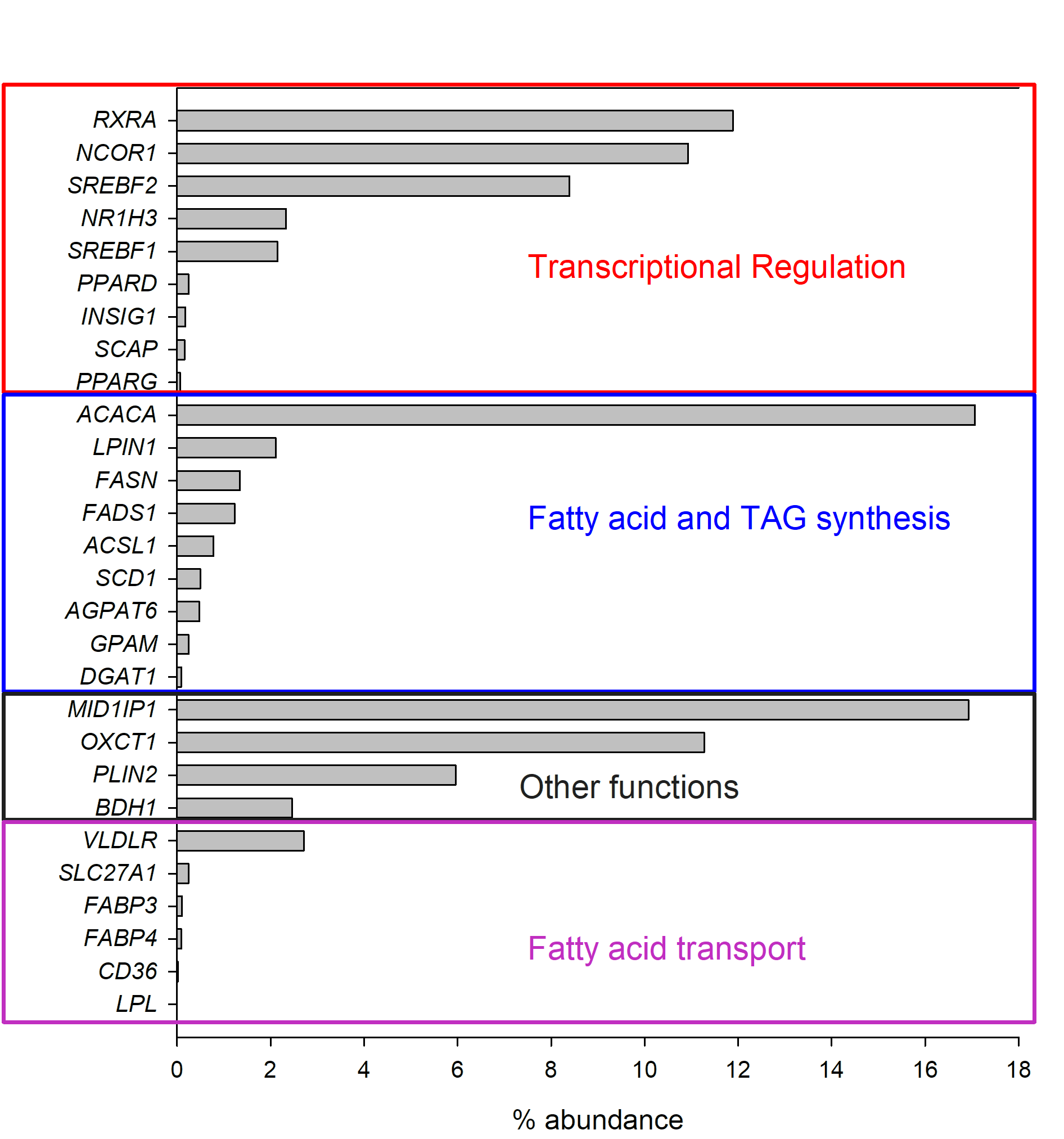

Supplement: Supplementary file 1 [file vetsci-06-00054-s001.zip › vetsci-497288-SI/Figure S1.TIF]

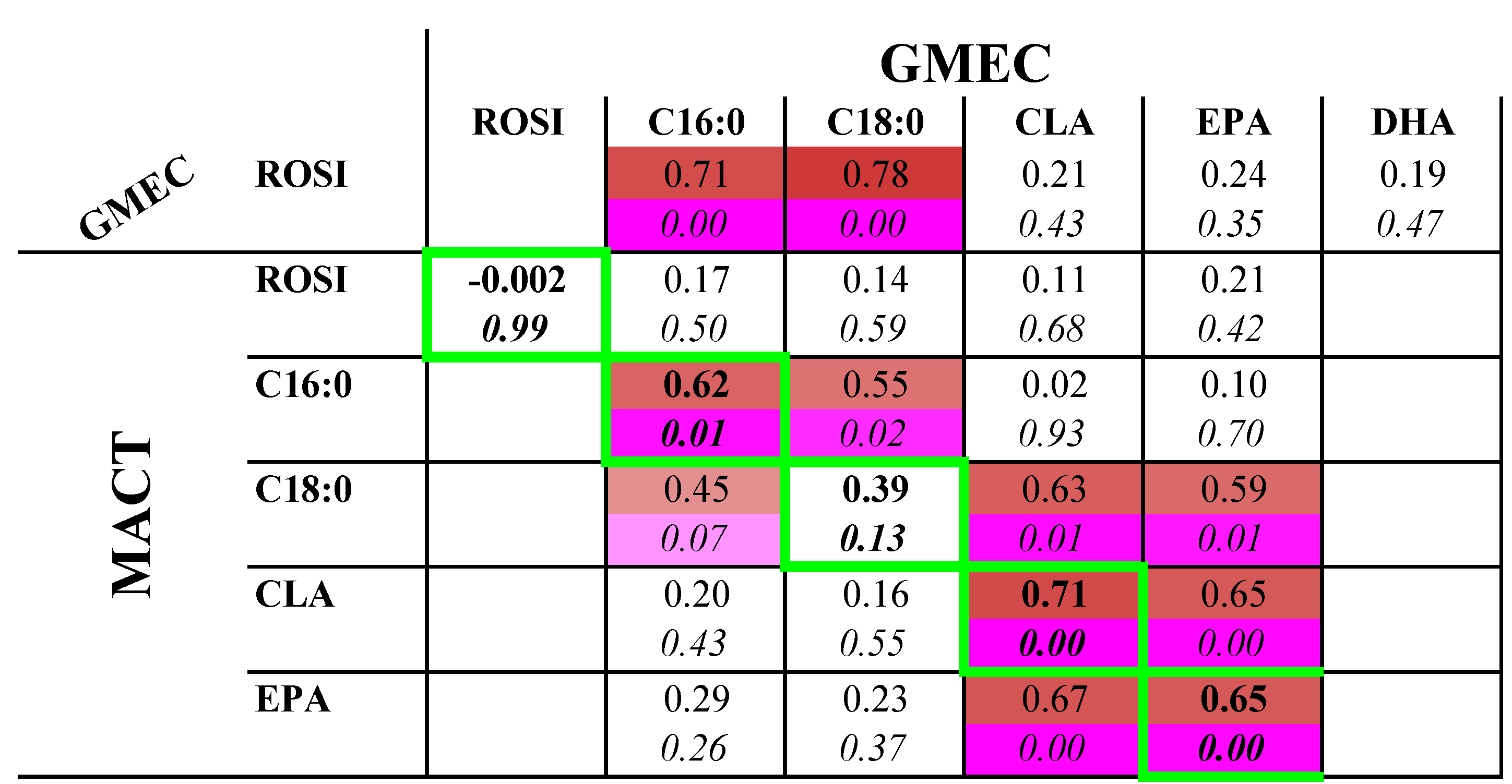

Supplement: Supplementary file 1 [file vetsci-06-00054-s001.zip › vetsci-497288-SI/Figure S2.tiff]

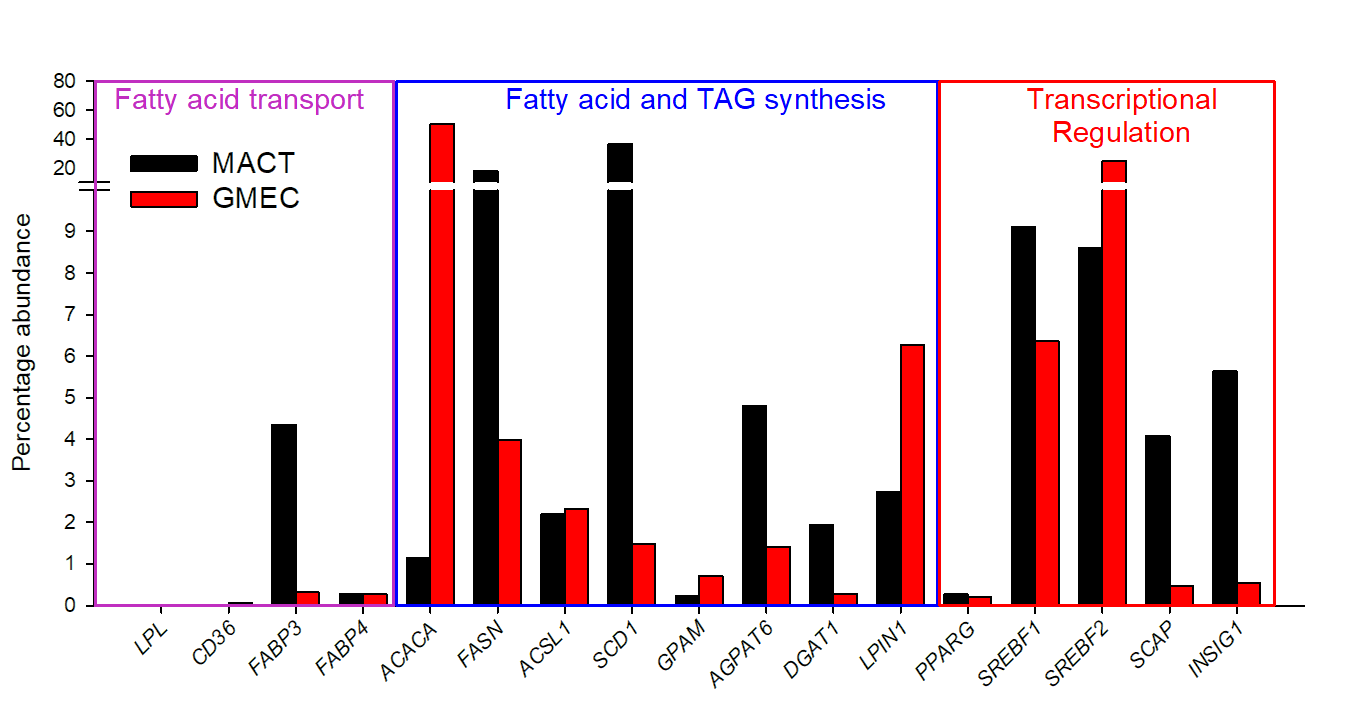

Supplement: Supplementary file 1 [file vetsci-06-00054-s001.zip › vetsci-497288-SI/Figure S3.tif]

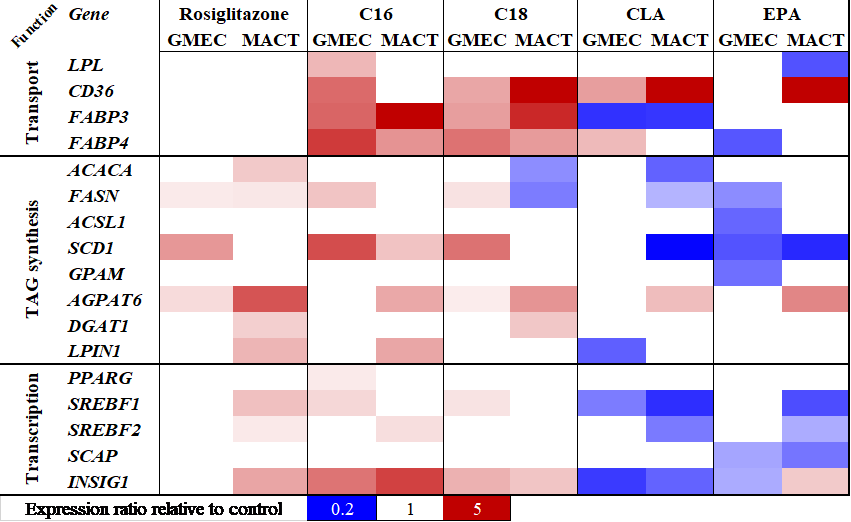

Supplement: Supplementary file 1 [file vetsci-06-00054-s001.zip › vetsci-497288-SI/Figure S4.tif]

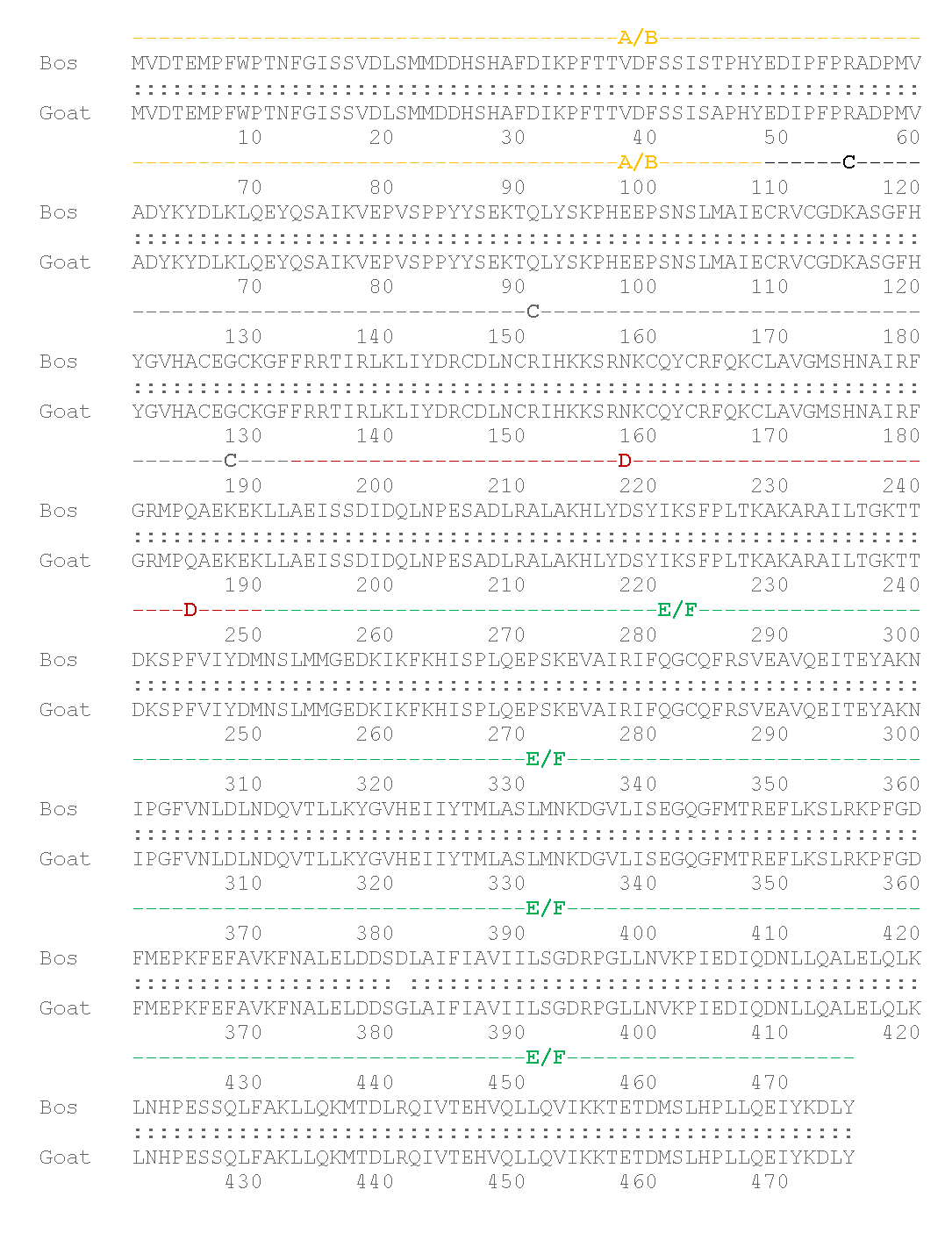

Supplement: Supplementary file 1 [file vetsci-06-00054-s001.zip › vetsci-497288-SI/Figure S5.tif]
